# A Rare Case of Unrecognized and Uncommon Bladder Perforation after Transobturator Tape Procedure

**DOI:** 10.1155/2015/731593

**Published:** 2015-01-05

**Authors:** Ercüment Kılınç, Yaşam Kemal Akpak

**Affiliations:** ^1^Department of Urology, Ankara Mevki Military Hospital, 06100 Ankara, Turkey; ^2^Department of Obstetrics and Gynecology, Ankara Mevki Military Hospital, 06100 Ankara, Turkey

## Abstract

The transobturator tape (TOT) procedure has become practically widespread worldwide. Complications seem to be rare, but recognizing them intraoperatively is the most significant step because some of the complications which may appear in postoperative period can be challenging for both physicians and patients. The purpose of this case, with this patient who was operated on with open surgery, is to evaluate this rarely seen unrecognized and uncommon bladder perforation after TOT procedure and thus make some contribution to the literature. Here, we present a case report about the treatment of a 48-year-old woman patient with unrecognized and uncommon bladder perforation after TOT procedure, 5 months postoperatively. Cystoscopic evaluation is not recommended routinely, but it must be performed if the patient is complicated enough to create doubt and also the surgeon's skill and ability are not sufficient enough to operate decently.

## 1. Introduction

Stress urinary incontinence (SUI), which has a major negative impact on the quality of life, is a widespread problem in women that will widely have operations in an effort to restore continence. Recently, various surgical procedures have been reported in literature. Midurethral slings have gradually become the gold standard surgical treatment for female SUI over the last years [1]. Unfortunately, these methods are not without complications, as in every surgical treatment. In particular, bladder perforation is a troubled complication, which may lead to morbidity, if left unrecognized. In a meta-analysis of retropubic and transobturator midurethral slings, bladder perforation occurrence rate was reported to be 6.7% [2]. According to our assessment, this is a significant rate and should be recognized in the surgery. Otherwise, open or endoscopic surgery would later be needed to remove the tape inside the bladder. We would like to share our experience of a patient with unusual and unrecognized bladder perforation and put emphasis on performing intraoperative cystoscopy to avoid such complications.

## 2. Case Report

**Figure 1:**
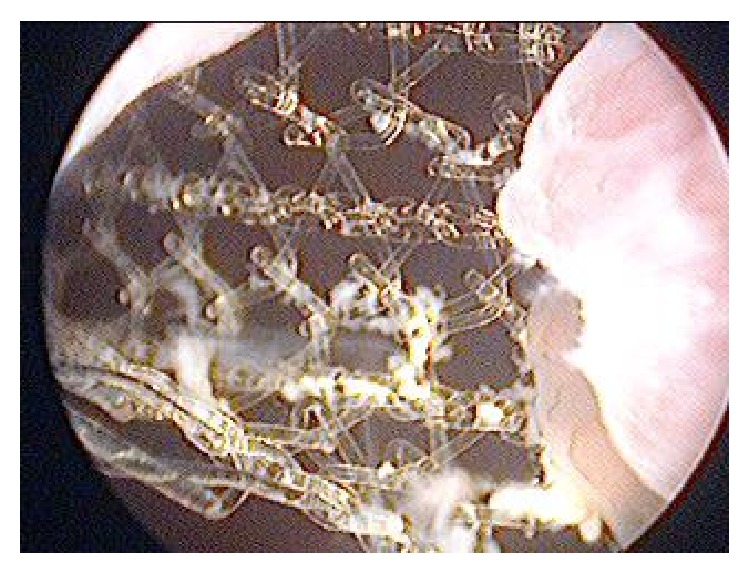
Cystoscopic view of the tape from urethra, perforating wall to wall of the bladder.

**Figure 2:**
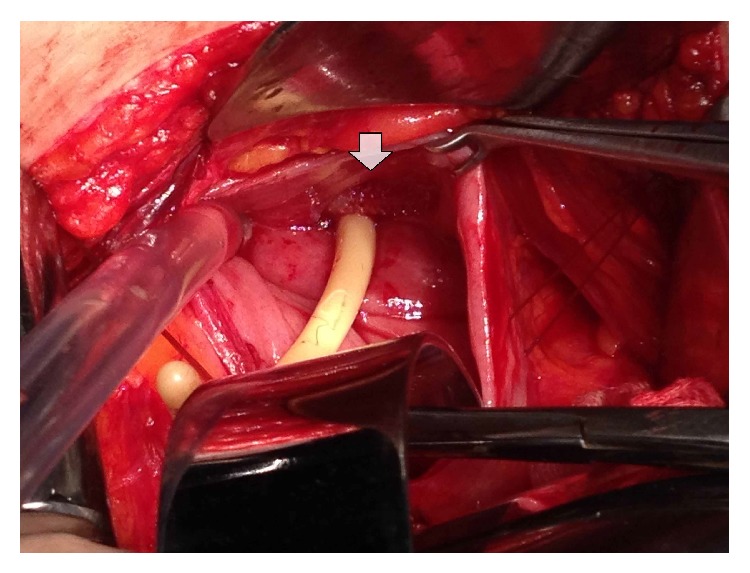
Open cystotomy through suprapubic approach, the mesh above the Foley catheter (under the arrow).

This patient, a 48-year-old woman, was admitted to our clinic with complaints of dysuria, frequency, urge incontinence, and suprapubic and pelvic pain that continued for 5 months. She had a history of a previous TOT procedure for stress urinary incontinence performed at another hospital 5 months ago. The patient had been discharged two days after surgery. The patient had haematuria for a while after the discharge. She had been admitted to many hospitals several times, including the first hospital with various symptoms during five months. Patient could not find enough interest from many doctors including initial doctors in her complaints, so she was admitted to our clinic. The patient did not have a detailed medical epicrisis. We tried to reach doctors who performed as the initial surgical procedure by phone repeatedly. Unfortunately, the surgical team consisting of a gynecologist and an urologist did not respond to our calls. It is understood from the patient's medical history that the procedure was done alone in the initial district hospital. In gynecological examination, we could not find any complications of previous surgery such fistula, infection, and erosion. Her weight was 52 kg, with a body mass index (BMI) of 21.6 kg/m^2^. She had no vaginal abnormalities. Leukocytes and erythrocytes were detected in the evaluation of urine sample. Enterococci grew in numbers of 80,000 colony-forming units per milliliter (cfu/mL) on urine culture at two different times. Appropriate treatment was held among the culture antibiotic sensitivity. Upon not responding to treatment cystoscopy was performed on patient under local anesthesia. In cystoscopic examination, a piece of mesh, about 3 cm length, extended from the right side of intravesical wall to the left side just behind the bladder neck (Figure 1). A bladder calculus was not determined on the exposed sling material. There were no gross pathologic findings of urethra, trigone, ureteral orifices, and bladder wall. Patient gave valid consent to surgical treatment after necessary information was given to her. Under general anesthesia, mini pfannenstiel incision was performed for suprapubic bladder incision. Anterior bladder wall was opened with vertical incision. The mesh, which was placed just behind the bladder neck, was attempted to be extracted with the help of clamp (Figure 2). Unfortunately, this attempt was not effective due to dense adhesions of mesh with extravesical tissue. The right and left lateral walls of the bladder associated with mesh were incised. Therefore, the intravesical part of mesh was carefully extracted under the mucosa and the muscular layer. At the first assessment, nature of the mesh was nonabsorbable material, typically polypropylene, and was constructed as a 2 cm wide mesh with a relatively large pore size. Extravesical material was left in its own place because of the dense fibrosis and adhesion. The defects of the detrusor muscle and mucosa were repaired with two layers using 3-0 polyglactin sutures (Vicryl, Ethicon, Johnson & Johnson, Brussels, Belgium). A 14 F three-way Foley catheter was inserted into the bladder. Bladder mucosa, muscle layer, and serosa were repaired and checked with watertight style. Bladder indwelling catheter was removed on the 7th postoperative day. The patient was discharged uneventfully. The patient was symptom-free at the follow-up visits 3 months after the operation.

## 3. Discussion

Midurethral slings for the treatment of stress urinary incontinence have been extensively practiced as a minimally invasive and effective surgery. In order to avoid the surgical complications, the transobturator approach was developed by Delorme in 2001 after the tension-free vaginal tape (TVT) [2]. Complications, including voiding difficulty, infection, rejection, and erosion of sling material rates of this procedure, are low. However, bladder perforation is one of the most widespread complications of the retropubic approach but using the transobturator route is more infrequent [1]. These injuries are associated with significant medical and medicolegal implications [3, 4]. In particular, most complications were observed between the first and fifth year of tape insertion [5]. In our case this situation was revealed five months later after the operation. The necessary experience as well as the skills is required to avoid bladder perforation in TOT surgeries. Of course the reasons are blind trocar passage and being more transverse during transobturator route [5]. If these complications are experienced for many reasons particularly inexperienced surgeons, cystoscopy will be the answer especially immediately after surgery for correcting misplaced sling material [6]. Also, the routine use of cystoscopy at the time of gynecologic surgery allows for timely diagnosis of urinary tract injuries in a cost-effective and cost-saving manner [7].

Surgical management of such complications, which were commonly located near bladder neck, has involved transurethral resection, open surgery, and cystoscopic and laparoscopic combined procedures [8]. However, some placements of mesh are very difficult for intervention with cystoscopy or not a technically feasible method for transurethral approach as in our case. You may not have enough visualization and space for intervention. Most patients can be managed with transvesical laparoscopic excision and reconstruction, but open surgery would be appropriate for adhesion formed mesh [8]. We chose open cystotomy through suprapubic approach to repair perfectly all layers of bladder including muscle and serosa layers and remove the entire mesh, which is unrecognized, distant from urethra, and adherent to the extravesical tissue due to dense adhesions and fibrosis, in the bladder.

Ultimately cystoscopic evaluation is not recommended routinely, but it must be performed if the patient is complicated enough to create doubt and also the surgeon's skill and ability are not sufficient enough to operate decently. If perforation can be determined immediately, it usually does not need any further therapy except catheter drainage for two or four days. Whichever the method chosen for bladder fixing is, mesh must be removed completely and should be repaired successfully.

## References

[1] Boyles S. H., Edwards R., Gregory W., Clark A. (2007). Complications associated with transobturator sling procedures. *International Urogynecology Journal and Pelvic Floor Dysfunction*.

[2] Latthe P. M., Foon R., Toozs-Hobson P. (2007). Transobturator and retropubic tape procedures in stress urinary incontinence: a systematic review and meta-analysis of effectiveness and complications. *BJOG*.

[3] Shepherd J. P., Rapkin R. B., Zyczynski H. M. (2012). Urethral injury with transobturator midurethral sling. *Female Pelvic Medicine and Reconstructive Surgery*.

[4] Dwyer P. L. (2010). Urinary tract injury: medical negligence or unavoidable complication?. *International Urogynecology Journal*.

[5] Petri E., Ashok K. (2012). Comparison of late complications of retropubic and transobturator slings in stress urinary incontinence. *International Urogynecology Journal and Pelvic Floor Dysfunction*.

[6] Dokmeci F., Yuce T., Cetinkaya S. E. (2014). Vesico-cutaneous fistula: unusual complication after transobturator mid-urethral sling. *International Urogynecology Journal*.

[7] Patel H., Bhatia N. (2009). Universal cystoscopy for timely detection of urinary tract injuries during pelvic surgery. *Current Opinion in Obstetrics and Gynecology*.

[8] Kim J. H., Doo S. W., Yang W. J. (2012). Laparoscopic transvesical excision and reconstruction in the management of midurethral tape mesh erosion andstones around the bladder neck: initial experiences. *BJU International*.

